# Deep-Ultraviolet Transparent Conductive MWCNT/SiO_2_ Composite Thin Film Fabricated by UV Irradiation at Ambient Temperature onto Spin-Coated Molecular Precursor Film

**DOI:** 10.3390/nano11051348

**Published:** 2021-05-20

**Authors:** Hiroki Nagai, Naoki Ogawa, Mitsunobu Sato

**Affiliations:** 1 Department of Applied Physics, School of Advanced Engineering, Kogakuin University, Tokyo 192-0015, Japan; nagai@cc.kogakuin.ac.jp; 2 Electrical Engineering and Electronics Program, Graduate School, Kogakuin University, Tokyo 192-0015, Japan; cm20014@ns.kogakuin.ac.jp

**Keywords:** deep ultraviolet, transparent, conductive thin film, MWCNT/SiO_2_, molecular precursor method, scratch resistance, heat and chemical resistance

## Abstract

Deep-ultraviolet (DUV) light-transparent conductive composite thin films, consisting of dispersed multiwalled carbon nanotubes (MWCNTs) and SiO_2_ matrix composites, were fabricated on a quartz glass substrate. Transparent and well-adhered amorphous thin films, with a thickness of 220 nm, were obtained by weak ultraviolet (UV) irradiation (4 mW cm^−2^ at 254 nm) for more than 6 h at 20−40 °C onto the precursor films, which were obtained by spin coating with a mixed solution of MWCNT in water and Si(IV) complex in ethanol. The electrical resistivity of MWCNT/SiO_2_ composite thin film is 0.7 Ω·cm, and transmittance in the wavelength region from DUV to visible light is higher than 80%. The MWCNT/SiO_2_ composite thin film showed scratch resistance at pencil hardness of 8H. Importantly, the resistivity of the MWCNT/SiO_2_ composite thin film was maintained at the original level even after heat treatment at 500 °C for 1 h. It was observed that the heat treatment of the composite thin film improved durability against both aqueous solutions involving a strong acid (HCl) and a strong base (NaOH).

## 1. Introduction

Deep-ultraviolet (DUV) light-transparent (<300 nm) conductive films are expected to improve the performance of various optoelectronic devices, including light-emitting diodes (LEDs) and solar cells. Although conventional conductive films such as indium-doped tin oxide (ITO) and fluorine-doped tin oxide (FTO) are the most widely used, the films absorb the DUV light owing to their bandgap energy. Therefore, a new type of transparent conductive film adaptable to a wide spectral range, including DUV light, is needed. Carbon nanotubes (CNTs) are of great interest for use as composites owing to their very high strength, electronic conductivity, and flexibility [1]. CNTs/inorganic composites have attracted interest in recent decades owing to their optical, mechanical, electrical, and thermal properties [2,3]. However, the poor adhesion of CNTs onto the substrate becomes a problem during usage. As a matrix of inorganic composites with CNTs, SiO_2_ is an ideal material because of its availability, chemical and thermal stability, and transparency to ultraviolet (UV) radiation. Accordingly, many researchers have reported nanocomposite powders of multiwalled carbon nanotubes (MWCNTs) with SiO_2_ powders using various techniques such as the sol–gel, electrophoretic deposition, and direct mixing of MWCNTs and SiO_2_ powders [2,4,5]. However, there are very few reports of MWCNT/SiO_2_ composite thin films fabricated by solution processing, because it is generally difficult to obtain uniformly dispersed MWCNTs with high content in the film due to their easy aggregation by strong van der Waals forces [3]. This suggests that a stable SiO_2_ precursor solution exhibiting high miscibility with MWCNT is required.

The molecular precursor method (MPM) is a wet chemical process that was developed by our research group for fabricating thin films of various metals, metal oxides, and phosphate compounds [6]. This method is based on the design of a metal complex, which has many advantages, such as excellent stability, homogeneity, and miscibility. Recently, the fabrication of crystallized Cu_2_O and amorphous titania thin films through UV irradiation of a precursor film was achieved by our research group [7,8]. The complexes used in the precursor films were successfully converted into functional thin films by irradiating UV light from a germicidal lamp at 30−40 °C.

In this study, we prepared a novel MWCNT/SiO_2_ precursor solution and fabricated a deep-ultraviolet transparent conductive MWCNT/SiO_2_ thin film well-adhered onto a quartz glass substrate by UV irradiation of the molecular precursor film. The electrical resistivity of the obtained MWCNT/SiO_2_ composite thin film was 0.7 Ω·cm. The composite thin film exhibited good thermal stability and antioxidation performance at 500 °C under atmospheric conditions. In addition, we reported the chemical stability of the UV-irradiated composite thin films and the effects of heat treatment on their durability against strong acids and bases in aqueous solutions.

## 2. Materials and Methods

### 2.1. Materials

Tetraethyl orthosilicate (TEOS) was purchased from Kanto Chemical Co., Inc. (Tokyo, Japan). Oxalic acid and sodium hydroxide (NaOH) were purchased from FUJIFILM Wako Pure Chemical Corporation (Miyazaki, Japan). Hydrochloric acid (HCl), 2-propanol, and 28% aqueous ammonia solution were purchased from Taisei Chemical Co., Ltd. (Tokyo, Japan). MWCNTs (2 wt %) dispersed in water (MWNT-INK, S_CNT_) were purchased from Meijo Nano Carbon Co., Ltd. (Aichi, Japan). Deionized water was purchased from Kyoei Pharmaceutical Co., Ltd. (Chiba, Japan). Ethanol (EtOH) was purchased from Ueno Chemical Industries, Ltd. (Tokyo, Japan) and dried on 4A molecular sieves prior to use. The other materials were used as received without further purification. Polished quartz glass was purchased from Akishima Glass Co., Ltd. (Tokyo, Japan). The quartz glass substrates (20 × 20 × 1.5 mm^3^) were prepared and cleaned using 2-propanol with an ultrasonic bath to remove organic molecules from the surfaces, followed by thorough rinsing with deionized water. The substrates were subsequently dried in an oven at 70 °C.

### 2.2. Preparation of SiO_2_ Precursor Solution (S_Silica_)

The SiO_2_ precursor solution (**S_Silica_**) was prepared by the reaction of 1.3 g of TEOS with 1.1 g of oxalic acid in 10 g of ethanol under reflux for 1 h. The concentration of Si^4+^ ions was 0.5 mmol g^−1^.

### 2.3. Preparation of MWCNT/SiO_2_ Composite Precursor Solution (S_COMP_)

The MWCNT/SiO_2_ composite precursor solution (**S_COMP_**) was prepared by mixing 0.1 g of **S_Silica_**, 0.3 g of MWNTs-INK, and 5.7 g of H_2_O at room temperature. Afterward, it was stirred for 1 h. 

### 2.4. Fabrication of MWCNT/SiO_2_ Composite Thin Films (F_COMP_) by UV-Light Irradiation and Heat Treatment (F’_COMP_)

The composite thin film was obtained as a three-layer film, as follows: 100 μL of **S_COMP_** was dropped on a quartz glass substrate of dimensions 20 × 20 mm^2^ using a micropipette, and subsequently spin-coated in double-step mode (first = 500 rpm for 5 s and second = 2000 rpm for 30 s). The precursor film was obtained by preheating the spin-coated film in a drying oven at 70 °C for 10 min. The first layer was formed by irradiating UV light at 254 nm (with a corresponding intensity of 4 mW cm^−2^) for 6 h onto the precursor film under a relative humidity range of 20−40% on a clean bench. The substrate surface temperature during UV irradiation (measured using a digital thermocouple) was in a range of 20−40 °C. The second layer was formed on the first layer by repeating the procedure used for the formation of the first layer. Thereafter, a well-adhered three-layer film on the quartz glass substrate was obtained by spin coating an additional layer of **S_Silica_** on the second layer, according to the procedure used for the first and second layers. The resultant thin film was denoted as **F_COMP_**. **F’_COMP_** was obtained by heat treating **F_COMP_** at 500 °C for 1 h in air using an electric furnace.

As a reference, a CNT single-layer thin film (**F_CNT_**) was formed by spin coating MWNT-INK and preheating using the abovementioned procedure. The **F’_CNT_** was obtained by heat treating the **F_CNT_** at 500 °C for 1 h in air using an electric furnace.

### 2.5. Optical Characterization of Solutions and Thin Films

The absorption spectra of the **S_Silica_** and TEOS solutions, whose Si^4+^ ion concentrations were adjusted to 0.5 mmol g^−1^ by dilution with ethanol, were measured in the wavelength region of 200–600 nm using a UV-1900i spectrophotometer (Shimadzu, Kyoto, Japan) in the double-beam mode. Ethanol was used as a reference. The transmittance spectra of **F_COMP_** and **F_CNT_** were measured in the range of 200–1100 nm in double-beam mode. A quartz glass substrate was used as a reference for the measurements.

### 2.6. Structural Characterization of Thin Films

The X-ray diffraction (XRD) patterns of **F_COMP_** and **F_CNT_** were measured using an X-ray diffractometer (SMART LAB, Rigaku, Tokyo, Japan) with Cu*-K*α rays generated at 45 kV and 200 mA. Parallel beam optics with an incident angle of 0.3° in the 2θ range of 10–80° and scanning at a 0.05° step width and speed of 5° min^−1^ were used (Supplementary Materials Figure S1). The Raman spectra of **F_COMP_**, **F’_COMP_**, **F_CNT_**, and **F’_CNT_** were measured using a Raman microspectrometer (LaBRAM HR800, Horiba Ltd., Kyoto, Japan) with a charge-coupled device detector. An Nd: YAG laser (532 nm) was used as the excitation source, with an intensity of 13 mW and an exposure time of 180 s. The spectra were measured in back-scattering geometry, and the spot diameter of the laser light was 1 μm. Spectra in the range of 900–2100 cm^−1^ were obtained by exposing the sample thin films to the laser beam for 30 min. Before the thin-film measurements, a silicon reference sample with a Raman peak at 520.64 cm^−1^ was used for the calibration of the wavelength. The Raman peaks of **F_COMP_**, **F’_COMP_**, **F_CNT_**, and **F’_CNT_** were quantitatively analyzed by using OriginPro2018b (OriginLab Corporation, Northampton, MA, USA), in order to evaluate the structural changes of MWCNT molecules by the heat-treatment. The peaks in the range of 900−1900 cm^−1^ were deconvoluted by a nonlinear least-square method with the use of Lorentz function. The peak fitting converged with the χ^2^ tolerance value of 1 × 10^−9^. The deconvoluted peaks are shown in the Supplementary Materials Figure S2.

### 2.7. Surface Morphologies, Film Thicknesses, and Pencil Hardnesses of F_COMP_ and F_CNT_

The surface morphologies of **F_COMP_** and **F_CNT_** were observed by field-emission scanning electron microscopy (FE-SEM) using a JSM-6701F microscope (JEOL Ltd., Tokyo, Japan) at an accelerating voltage of 5 kV. The average grain size of each thin film was calculated from 10 randomly selected grains.

The thicknesses of the thin films were measured using a stylus profilometer (DEKTAK-3, Sloan, CA, USA). For sample preparation, a portion of each precursor film was removed by ethanol to expose the substrate. The level differences at five positions between the substrate and the resultant thin film were measured for each sample. The film thickness was calculated as the average value, excluding the highest and lowest values.

The pencil hardness was evaluated according to the Japanese Industrial Standard (JIS) K5400 by a pencil scratch test using an MJ-PHT pencil hardness meter (Sato Shouji Inc., Kanagawa, Japan) with a 0.75 kg load. The film was scratched using a pencil (UNI, Mitsubishi Pencil Co., Ltd., Tokyo, Japan) with various hardness values standardized in the hardening order from 6B to 9H.

### 2.8. Electrical Resistivities of F_COMP_, F’_COMP_, F_CNT_, and F’_CNT_

The electrical resistivities of the thin films were measured using the four-probe method involving two multimeters (VOAC7512, Iwatsu and Model Multimeter, Keithley, Fort Worth, TX, USA) and a regulated DC power supply (Model PAB 32-1.2, Kikusui Electronics Corp., Yokohama, Japan) at 25 °C. Four gold-plated tungsten probes (FELL type, K&S, Washington, DC, USA) were placed at intervals of 1 mm, and a load of 0.1 kg was applied.

### 2.9. Chemical Stabilities of F_COMP_ and F’_COMP_ against Acids and Bases in Water

Three samples of **F_COMP_** were prepared and each sample was immersed in a 10% aqueous solution of hydrochloric acid (HCl, strong acid; sa), ammonia (NH_3_, weak base; wb), and sodium hydroxide (NaOH, strong base; sb) for 1 h at 20 °C. The immersed samples in each solution were rinsed with water and dried at 70 °C for 10 min, and denoted as **F_COMP_-**sa, **F_COMP_-**wb, and **F_COMP_-**sb, respectively. Additionally, three samples of **F’_COMP_** were prepared and treated in procedures identical to those used for **F_COMP_**. The obtained samples were denoted as **F’_COMP_-**sa, **F’_COMP_-**wb, and **F’_COMP_-**sb, respectively. The film thicknesses, electrical resistivities, and hardnesses of these films were measured to evaluate their chemical stabilities.

## 3. Results and Discussion

### 3.1. Absorption Spectra of S_Silica_

**Figure 1:**
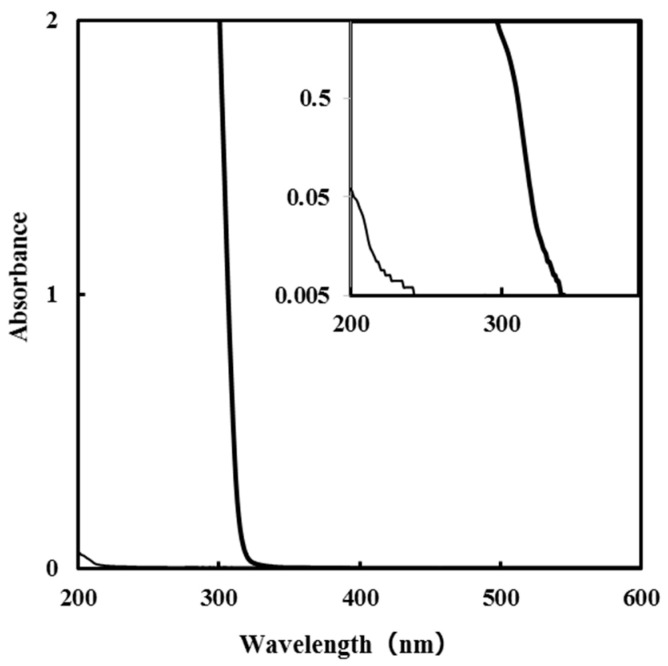
Absorption spectra of the **S_Silica_** (bold line) and Tetraethyl orthosilicate (TEOS) solutions in ethanol (thin line). The concentrations (0.5 mmol g^−1^) of Si^4+^ ions are identical to each other. The inset plotted the absorbance on a semi-log scale.

The absorption spectra of the **S_Silica_** and TEOS solutions of 0.5 mmol g^−1^ in ethanol are shown in Figure 1. The absorption of **S_Silica_** observed in the UV region is stronger than that of the TEOS solution, although the concentrations of Si^4+^ ions in both solutions are equal.

Some researchers have reported the tris (oxalato) complex of Si^4+^ ions [9,10]. The strong absorption in the UV-light region of **S_Silica_** indicates that identical coordination bonds are formed by the reaction of TEOS and oxalic acid in this precursor solution. To obtain high solubility in ethanol by assuming a neutral Si complex, the molar amount of the added oxalic acid was two times higher than that of the Si^4+^ ion. As a result, a precursor solution with excellent miscibility and coatability could be prepared.

We previously reported the fabrication of amorphous titania thin films by applying a low-power germicidal lamp with an irradiation peak at 254 nm, for 4 h under a relative humidity range of 40–60% at 30–40 °C [8], onto the precursor film comprising the titanium complex of an oxalato ligand. The oxalato ligand can be removed stepwise by UV irradiation. This is because of the strong absorption which can be attributed to the charge transfer (CT) transition band in the UV-light region, as shown in Figure 1.

### 3.2. Structural Characterization of F_COMP_ and F’_COMP_

**Figure 2:**
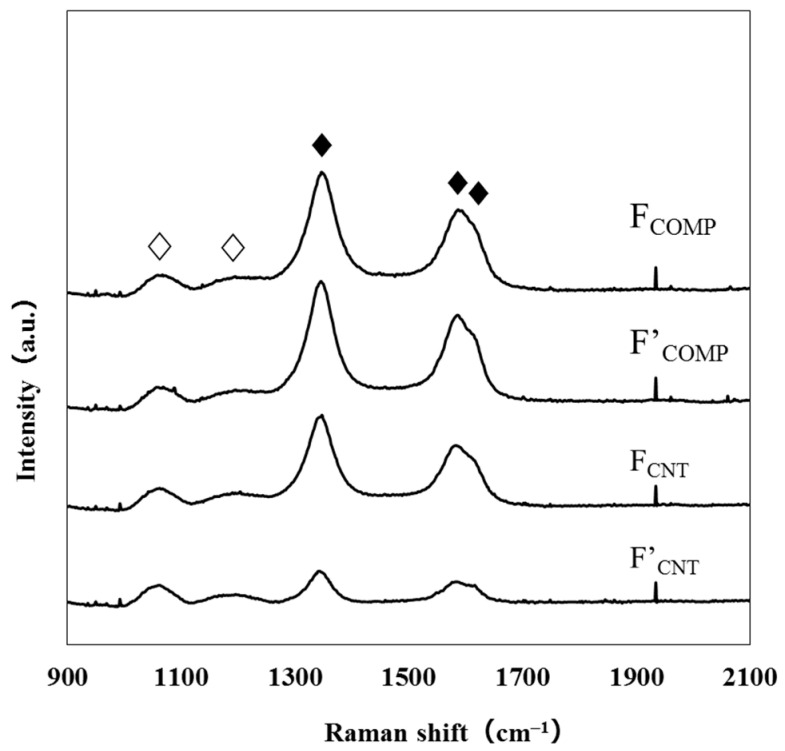
Raman spectra of the resultant thin films. The peaks attributable to the vibration modes for CNT and SiO_2_ are represented by ♦ and ♢, respectively.

Several researchers reported that the periodically aggregated MWCNTs indicate XRD peaks at 2θ = 26 and 43°, assignable to the (002) and (100) phases [11,12,13]. However, XRD patterns of **F_COMP_** and **F_CNT_** show no peak, indicating the randomly arranged MWCNTs in amorphous SiO_2_ matrix (Supplementary Materials Figure S1). Figure 2 shows the Raman spectra of **F_COMP_**, **F’_COMP_**, **F_CNT_**, and **F’_CNT_**. The two peaks at 1060 and 1190 cm^−1^ are the transverse optic (TO) and the longitudinal optic (LO) ω4 modes of SiO_2_, respectively [14]. The three peaks at 1350, 1590, and 1620 cm^−1^ can be ascribed to the D, G, and D’ bands of CNT [15]. Totally five peaks could be successfully deconvoluted (Suppplementary Figure S2). Each peak area of the deconvoluted peaks was normalized with that of the TO band of SiO_2_. As a result, the (D + D’)/TO and G/TO ratios of **F_CNT_** decreased by 71% and 62%, respectively, by the heat treatment to produce **F’_CNT_**, indicating the significant loss of the MWCNT molecules. On the other hand, those ratios of **F_COMP_** decreased by only 12% and 4%, respectively. These results clearly indicate that the SiO_2_ matrix plays an important role to prevent the co-present MWCNT molecules from oxidation. However, it is acceptable that the slightly increased resistivity of **F’_COMP_** as compared to **F_COMP_** is owing to the decrease of MWCNT molecules in the film (see Section 3.4, Supplementary Materials Table S1). It was additionally revealed that the defective parts of CNTs are more likely to be lost by the heat treatment at 500 °C for 1 h, than the graphite parts.

### 3.3. Surface Morphologies of F_COMP_ and F_CNT_

**Figure 3:**
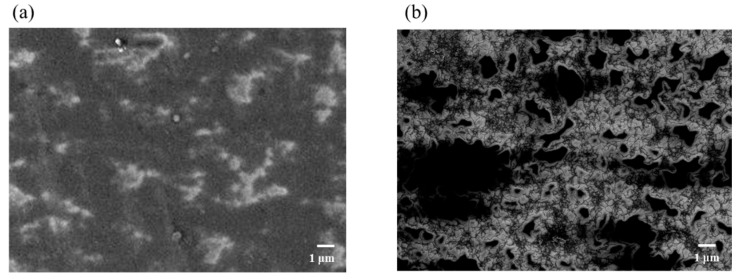
FE-SEM images of (**a**) **F_COMP_** (**b**) **F_CNT_**.

Figure 3 shows the surface morphologies of **F_COMP_** and **F_CNT_**, which are observed using FE-SEM. The surface morphology of **F_COMP_** suggests the dispersion of MWCNT molecules in the SiO_2_ matrix, though the corresponding molecules are clearly observed in that of **F_CNT_**. The SiO_2_ precursor solution was highly miscible with the dispersed solution of MWCNT. Therefore, the MWCNT molecules did not aggregate by van der Waals forces in the mixed solution with **S_Silica_**, and a homogeneous film could be obtained.

### 3.4. Film Thicknesses, Pencil Hardnesses, and Electrical Resistivities of F_COMP_ and F’_COMP_, and Their Chemical Stabilities

Table 1 summarizes the film thicknesses, electrical resistivities, and pencil hardnesses of the obtained three-layer composite thin films. Before coating with the SiO_2_ thin film, the one- and two-layer thin films were softer than the pencil hardness of 6B. However, the thin film **F_COMP_** became harder than 8H after it was coated with SiO_2_ as the third layer, which fully covered the composite film, as shown in the SEM image (Figure 3a). It is important to note that the resistivity of the heat-treated **F’_COMP_** can be maintained at the original level after heat treatment at 500 °C under atmospheric conditions for 1 h, and the decrease in film thickness was within 5%.

Conversely, the film thickness of the **F_CNT_** could not be measured using the stylus profilometer, because the MWCNT film was easily peeled off the substrate by tracing the surface with the stylus. For this reason, the electrical conductivity of **F_CNT_** could not be determined using the four-probe method.

To examine the chemical stability of the obtained thin films, the **F_COMP_** was immersed separately in two 10% aqueous solutions of a strong acid (HCl) and a strong base (NaOH) for 1 h at 20 °C. As a result, the film thickness of the obtained **F_COMP-_**sa after immersion in the 10% HCl solution decreased by approximately 18%, which is larger than that of the **F’_COMP_** obtained by heat treatment at 500 °C for 1 h. However, the electrical resistivity of **F_COMP-_**sa was almost identical to that of **F’_COMP_**. The obtained **F_COMP_** was peeled off by immersion in the 10% NaOH solution. Therefore, a 10% aqueous ammonia solution was used to test the durability of the **F_COMP_** against a weak base. The obtained thin film **F_COMP-_**wb exhibited an identical film thickness and electrical resistivity as **F_COMP-_**sa, although the pencil hardness was inferior to those of the other treated films. All the film thickness of composite thin films decreased in almost identical levels, 18−19%, with the chemical treatment. It can be assumed that the dangling bonds of incomplete Si–O network on the surface of the composite thin films after UV irradiation exist and can react with acids and bases. As mentioned above, the slight shrinkage of the film thickness by the heat treatment of **F_COMP_** occurred. It is thus clear that the heat treatment in air contributed to form more rigid Si−O bond network. The chemical stability of **F’_COMP_** against basic aqueous solutions became to be higher than that of **F_COMP_**, because the dangling bonds in Si–O network are presumed to be reactive highly with the employed bases. It is very important to note that the heat-treated **F’_COMP_** is highly robust in both aqueous solutions involving strong acids (HCl) and bases (NaOH), producing **F’_COMP-_**sa and **F’_COMP-_**sb, respectively.

### 3.5. Optical Properties of the F_COMP_ and F_CNT_

**Figure 4:**
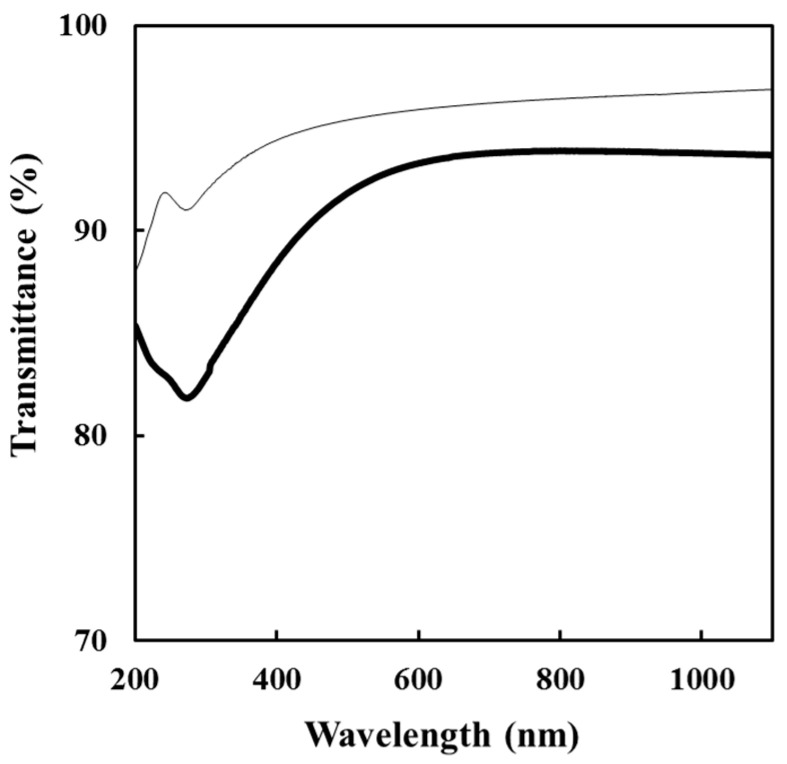
Transmittance spectra of **F_COMP_** (bold line) and **F_CNT_** (thin line).

Figure 4 shows the transmittance spectra of **F_COMP_** and **F_CNT_**. The **F_COMP_** exhibited more than 80% transmittance in the wavelength region from DUV to visible light. The characteristic absorption at 273 nm (4.5 eV) is due to the π-plasmon of carbon nanotubes. The absorption peaks are related to the polarization dependence and the optical properties of graphite [16]. Lehman et al. reported that MWCNTs exist as aggregates and bundles in suspension, with no special features in the UV and visible spectra. If the sonication energy is sufficient to overcome the van der Waals attraction between adjacent tubes in the solution, the entanglement is undone and dispersibility increases, which is associated with more obvious spectral characteristics, such as the appearance of π-plasmon peaks [17]. The plasmon peak of **F_COMP_** is stronger than that of **F_CNT_**. This indicates that the MWCNT molecules in the **F_COMP_** distribute more homogeneously than those in the **F_CNT_**. It is consistent that the FE-SEM images demonstrate the homogeneous distribution of MWCNT molecules, which leads to a low electrical resistivity of the composite film.

## 4. Conclusions

A SiO_2_ precursor solution with no precipitation was prepared by forming the Si^4+^ complex of oxalic acid in the molar ratio of 1:2 in ethanol. The SiO_2_ precursor solution was highly miscible with an aqueous solution dispersed of MWCNTs. The mixed solution was adequate to form a homogenous precursor film, which can be easily converted to a MWCNT/SiO_2_ composite thin film by irradiating UV light from a germicidal lamp via a spin-coating process on a quartz glass substrate. Thus, the MPM used in the present study is an effective and facile method for the low-temperature fabrication of conductive thin films with transparency in the wavelength region from DUV to visible light. This unprecedented thermally and chemically robust composite thin film may improve the performance of various optoelectronic devices, including LEDs and solar cells.

## Figures and Tables

**Table 1:** Film thicknesses, electrical resistivities, and pencil hardnesses of **F_COMP_** and **F’_COMP_** fabricated onto the quartz glass substrate, along with those of the chemically treated thin films **F_COMP-_**sa, **F’_COMP-_**sa, **F_COMP-_**wb, **F’_COMP-_**wb, and **F’_COMP-_**sb.

Film	Film Thickness	Electrical Resistivity	Pencil Hardness
	nm	Ω·cm	
**F_COMP_**	220	0.7 ± 0.1	8H
**F’_COMP_**	210	0.9 ± 0.1	8H
**F_COMP-_**sa	180	0.7 ± 0.1	8H
**F’_COMP-_**sa	180	0.9 ± 0.1	8H
**F_COMP-_**wb	180	0.8 ± 0.1	4H
**F’_COMP-_**wb	180	0.9 ± 0.1	8H
**F’_COMP-_**sb	170	0.8 ± 0.1	8H

## Data Availability

Not applicable.

## Supplementary Materials

The following are available online at https://www.mdpi.com/article/10.3390/nano11051348/s1., Figure S1 XRD patterns of the obtained thin films, (a) **F_COMP_**, (b) **F’_COMP_**, (c) **F_CNT_**, and (d) **F’_CNT_**., Figure S2: Deconvoluted Raman peaks of the obtained thin films, (a) **F_COMP_**, (b) **F’_COMP_**, (c) **F_CNT_** and (d) **F’_CNT_**, along with each original spectrum in the range of 900–1900 cm^–1^. The thick line indicates the observed spectra, while five lines, (- - -), (— — —), (• • •), (—), and (— • —) indicate the theoretically fitted curves assignable the TO, LO, D, G, and D’ bands, respectively. All the calculated curves completely overlapped the corresponding observed curves., Table S1; The (D+D’)/TO and G/TO peak area ratios calculated from the Raman spectra of the obtained thin films, along with the decreasing levels of the ratios with the heat treatment are shown. The peak area was normalized with the TO band of SiO_2_.
