# Supplementary material for: Deep-Ultraviolet Transparent Conductive MWCNT/SiO2 Composite Thin Film Fabricated by UV Irradiation at Ambient Temperature onto Spin-Coated Molecular Precursor Film
Source: Nanomaterials (Basel). 2021 May 20;11(5):1348. doi: 10.3390/nano11051348 (PMC8161176; doi:10.3390/nano11051348)
Supplement: Supplementary file 1 [file nanomaterials-11-01348-s001.zip › nanomaterials-1198222-supplementary.pdf]

Supplementary Materials

Deep-Ultraviolet Transparent Conductive MWCNT/SiO_2 Composite Thin Film Fabricated by UV Irradiation at Ambient Temperature onto Spin-Coated Molecular Precursor Film

Hiroki Nagai [1], Naoki Ogawa [2] and Mitsunobu Sato [1,*]

[1] Department of Applied Physics, School of Advanced Engineering, Kogakuin University, Tokyo 192-0015, Japan; nagai@cc.kogakuin.ac.jp (H.N.)
[2] Electrical Engineering and Electronics Program, Graduate School, Kogakuin University, Tokyo 192-0015, Japan; cm20014@ns.kogakuin.ac.jp (N.O.)
* Correspondence: lccsato@cc.kogakuin.ac.jp; Tel.: +81-426-731-492

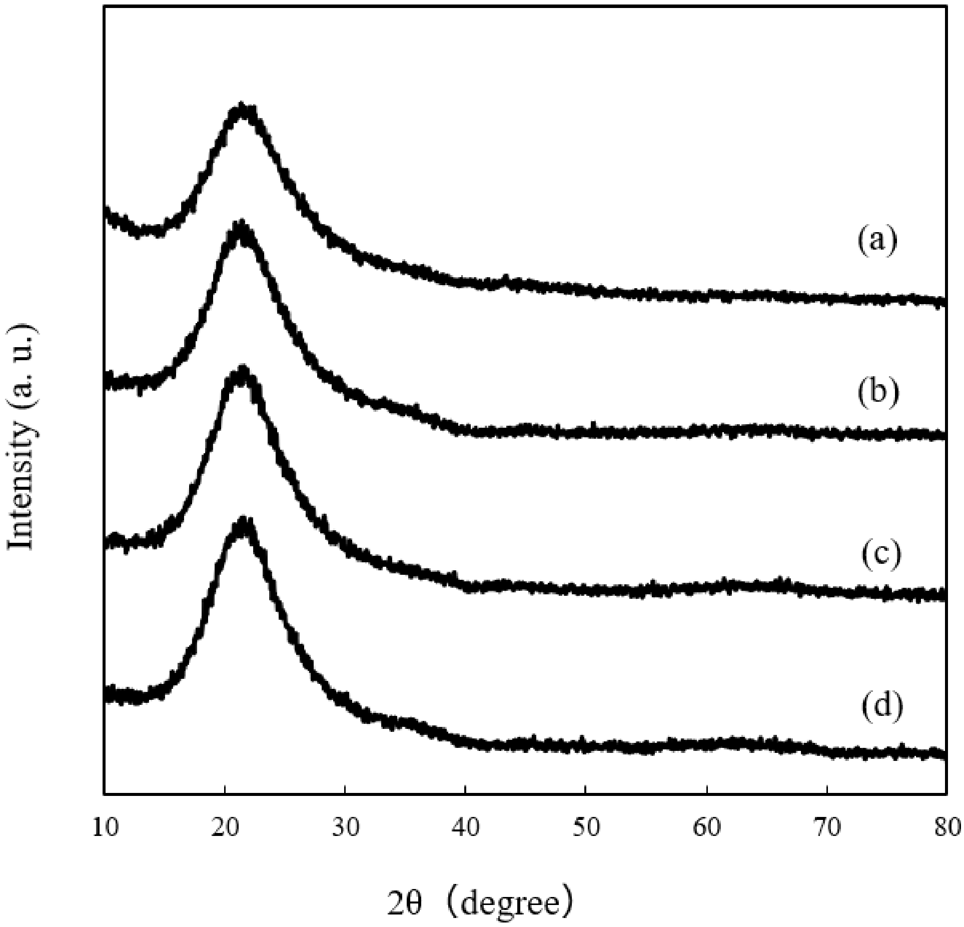

Figure S1. XRD patterns of the obtained thin films, (**a**) **F_{COMP}**, (**b**) **F'_{COMP}**, (**c**) **F_{CNT}**, and (**d**) **F'_{CNT}**.

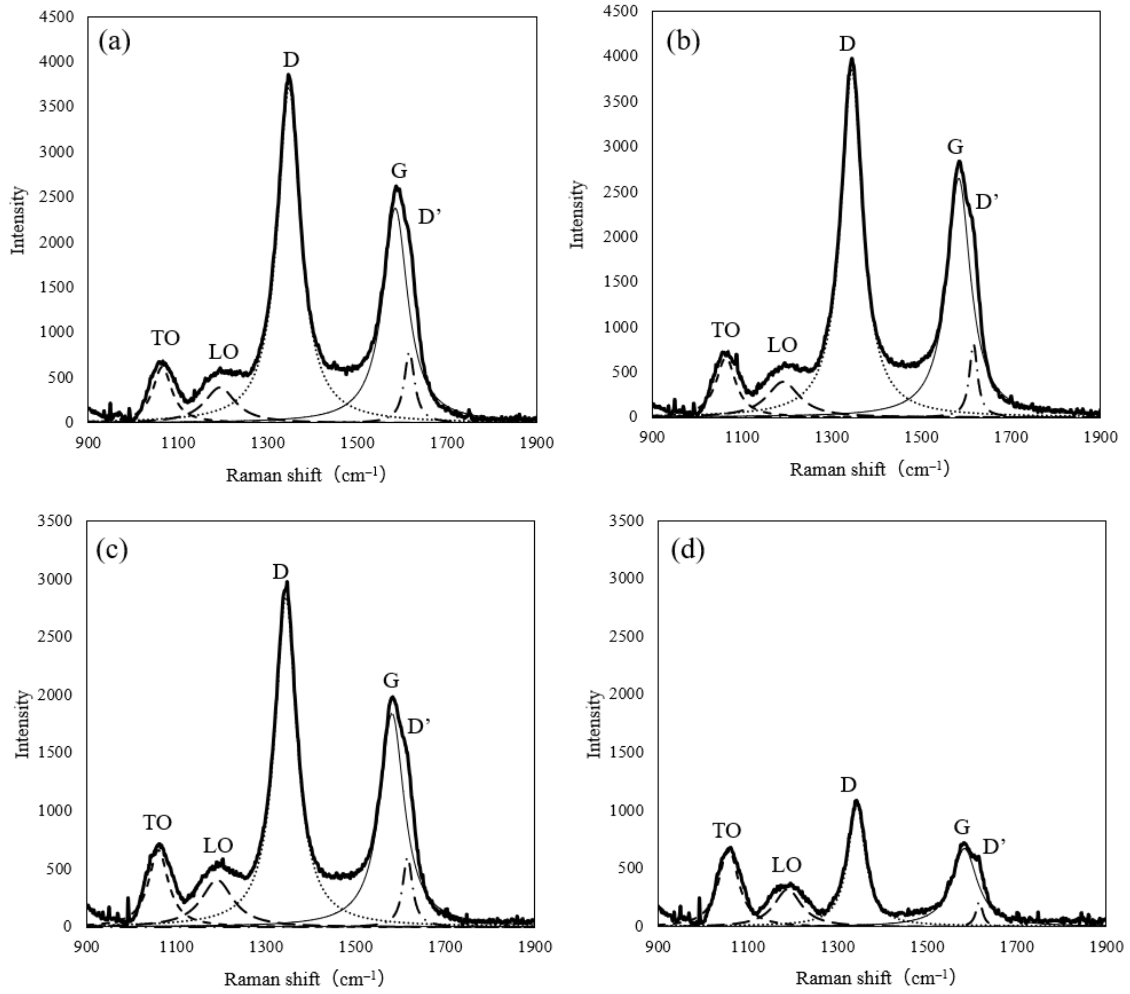

Figure S2. Deconvoluted Raman peaks of the obtained thin films, (**a**) $\mathbf{F}_{\mathbf{COMP}}$, (**b**) $\mathbf{F'}_{\mathbf{COMP}}$, (**c**) $\mathbf{F}_{\mathbf{CNT}}$ and (**d**) $\mathbf{F'}_{\mathbf{CNT}}$, along with each original spectrum in the range of 900–1900 cm^{-1}. The thick line indicates the observed spectra, while five lines, (- - -), (— — —), (• • •), (—), and (— • —) indicate the theoretically fitted curves assignable the TO, LO, D, G, and D′ bands, respectively. All the calculated curves completely overlapped the corresponding observed curves.

Table S1. The (D+D′)/TO and G/TO peak area ratios calculated from the Raman spectra of the obtained thin films, along with the decreasing levels of the ratios with the heat treatment are shown. The peak area was normalized with the TO band of SiO_2.

Films	(D+D')/TO	Decreasing level by heat treatment	G/TO	Decreasing level by heat treatment
F_{COMP}	8.1		5.0	
		12%		4%
F'_{COMP}	7.1		4.8	
F_{CNT}	5.2		3.4	
		71%		62%
F'_{CNT}	1.5		1.3	
